# H-Ras Expression in Immortalized Keratinocytes Produces an Invasive Epithelium in Cultured Skin Equivalents

**DOI:** 10.1371/journal.pone.0007908

**Published:** 2009-11-19

**Authors:** Melville B. Vaughan, Ruben D. Ramirez, Capri M. Andrews, Woodring E. Wright, Jerry W. Shay

**Affiliations:** 1 Department of Cell Biology, University of Texas Southwestern Medical Center, Dallas, Texas, United States of America; 2 Department of Biology, University of Central Oklahoma, Edmond, Oklahoma, United States of America; Roswell Park Cancer Institute, United States of America

## Abstract

**Background:**

Ras proteins affect both proliferation and expression of collagen-degrading enzymes, two important processes in cancer progression. Normal skin architecture is dependent both on the coordinated proliferation and stratification of keratinocytes, as well as the maintenance of a collagen-rich basement membrane. In the present studies we sought to determine whether expression of H-ras in skin keratinocytes would affect these parameters during the establishment and maintenance of an *in vitro* skin equivalent.

**Methodology/Principal Findings:**

Previously described cdk4 and hTERT immortalized foreskin keratinocytes were engineered to express ectopically introduced H-ras. Skin equivalents, composed of normal fibroblast-contracted collagen gels overlaid with keratinocytes (immortal or immortal expressing H-ras), were prepared and incubated for 3 weeks. Harvested tissues were processed and sectioned for histology and antibody staining. Antigens specific to differentiation (involucrin, keratin-14, p63), basement-membrane formation (collagen IV, laminin-5), and epithelial to mesenchymal transition (EMT; e-cadherin, vimentin) were studied. Results showed that H-ras keratinocytes produced an invasive, disorganized epithelium most apparent in the lower strata while immortalized keratinocytes fully stratified without invasive properties. The superficial strata retained morphologically normal characteristics. Vimentin and p63 co-localization increased with H-ras overexpression, similar to basal wound-healing keratinocytes. In contrast, the cdk4 and hTERT immortalized keratinocytes differentiated similarly to normal unimmortalized keratinocytes.

**Conclusions/Significance:**

The use of isogenic derivatives of stable immortalized keratinocytes with specified genetic alterations may be helpful in developing more robust in vitro models of cancer progression.

## Introduction

Two major types of skin cancer, squamous cell carcinoma and basal cell carcinoma, affect the primary skin epidermal cell type termed the keratinocyte. While basal cell carcinomas locally invade, the ability of squamous cell carcinomas to migrate and spread to other areas of the body is of great concern. One of the hallmarks of metastatic carcinoma cells is the epithelial to mesenchymal transition (EMT), characterized by changes in the cytoskeleton and cell-to-cell connections [1]–[2] including decreased E-cadherin and increased vimentin expression. These protein changes primarily affect the integrity of the stratified epithelial cells' attachment to each other. However, extracellular changes will disrupt the entire architecture. Inappropriate proliferation and extracellular matrix degradation produce a tissue mosaic instead of a compartmentalized, stratified epithelium. While it appears that there are many cellular changes necessary to produce a skin carcinoma, at least two major pathways are likely to be involved [3]. One such pathway involves Ras activation signaling [4]; specifically, H-Ras has been shown to affect proliferation [5] and matrix degradation [6]–[7]. While it remains unknown if a partial or complete EMT program underlies the invasive/metastatic phenotype of all high-grade human tumors, cell-based assays such as those presented here may permit addressing this central question.

The normal histological appearance of human epidermis is that of a stratified epithelium, composed primarily of keratinocytes, situated atop a collagen IV-rich basement membrane. The basal keratinocyte layer is unique in that it is characterized by keratin-14 and p63 expression. Protein expression patterns in the suprabasal layers change as keratinocytes commit to producing a barrier; basal proteins disappear and suprabasal proteins such as keratin-10 and involucrin become apparent [8]–[10]. During cancer progression, keratinocytes escape from their compartment and migrate into new tissues, where they may interact with their new environment [11].

**Figure 1:**
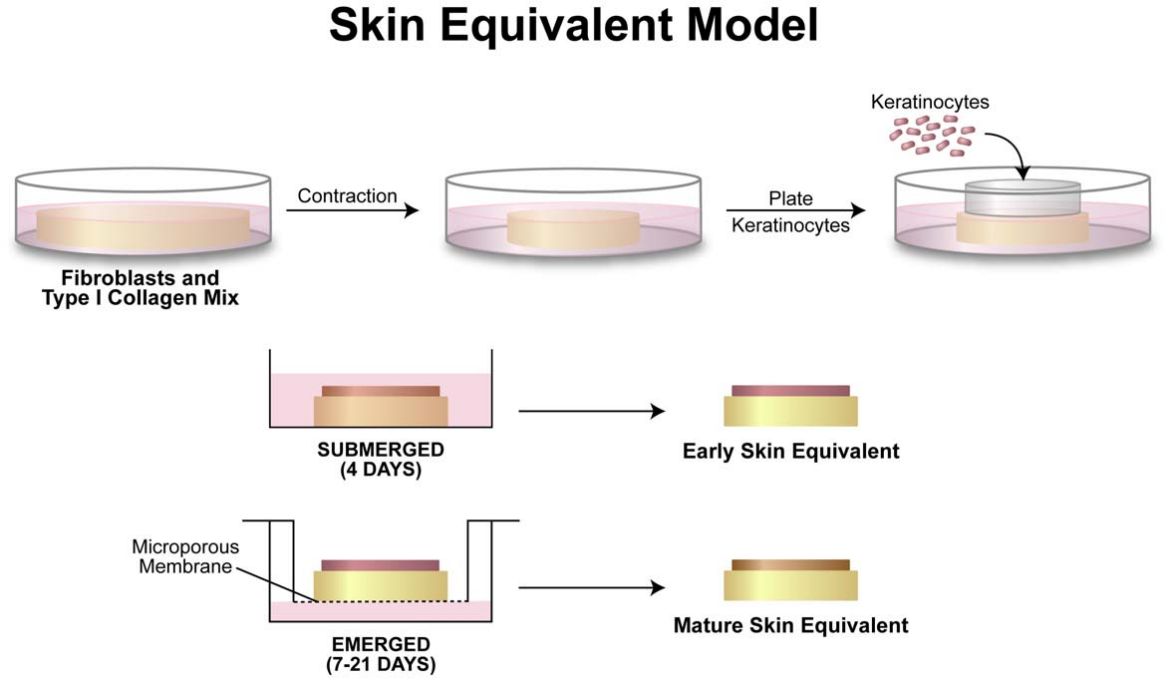
Diagram of the skin equivalent model. Fibroblasts were mixed with collagen and allowed to contract over a period of 4–7 days. Keratinocytes were then plated, using a cloning ring, at a density of 200,000 cells/cm^2^. The cells were allowed 4 hours to settle and attach to the upper surface of the contracted collagen lattice. After 4 days of submerged culture, the skin equivalents were raised to the air/liquid interface through subsequent culturing in the upper chamber of a Transwell™ plate. Skin equivalents of emerged cultures were harvested at 7, 14, and 21 days.

The complexities of studying cancer progression in animal models have demonstrated the need for more simplified organotypic models to study mechanisms of differentiation and cancer progression. New immortalization techniques using telomerase (hTERT) combined with better culturing methods are providing new cell-based models for cell differentiation and cancer progression studies [12]–[14]. In this study we have used the skin organotypic culture environment to mimic a simplified human skin equivalent in vitro [15]–[16]. The skin equivalent (Figure 1) is composed of fibroblasts, collagen, and keratinocytes, and similar to skin, the epithelium will stratify and differentiate [17]. In addition to serving as an experimental model for studying skin development, wound healing, aging skin and other circumstances where the basement membrane may be compromised, another potential benefit of this model is the ability to demonstrate dermal invasion by the epithelium and provide information on how epithelial organization is affected in the presence of oncogenic changes or by experimental manipulations such as exposure to terrestrial or space radiation [18].

This present study was performed to determine whether ectopic H-Ras expression, with or without p53 inhibition, was sufficient to induce transformation of immortalized keratinocytes in a skin equivalent organotypic culture model system [12]. In addition, cells derived from H-Ras keratinocytes that formed tumors in mice were compared in the skin equivalent model (Table 1). The results demonstrated subtle but increasing EMT while more dramatic changes were seen in tissue architecture, initially in the lower epithelium but extending throughout the epithelium as a result of subsequent experimental cellular modifications.

**Table 1:** Keratinocytes used in this study, with abbreviations.

Cell Name	Description
Ker-CT	Normal foreskin keratinocytes immortalized with cdk4 and human telomerase [12]
Ker-CT-Ras	Above cells with H-Ras
Ker-CT-Ras-p53	Ker-CT-Ras with a mutant p53 (His273)
Ker-CT-Ras-T	Tumor-forming Ker-CT-Ras

## Results

### Keratinocytes That Express Ectopically Introduced H-Ras Lose Normal Stratification and Invade into the Dermal Compartment

**Figure 2:**
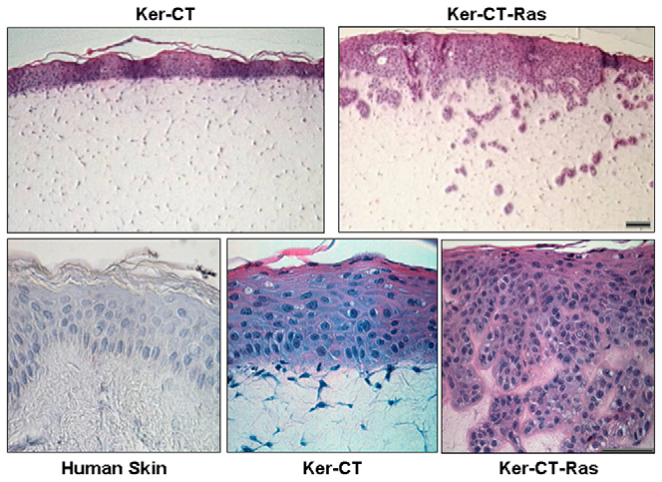
Ker-CT-Ras keratinocytes produced a randomized, invasive epithelium. While Ker-CT keratinocytes remain as a surface epithelium on the skin equivalent (upper left), Ker-CT-Ras keratinocytes appear to invade the dermal compartment (upper right). At higher magnification, normal human skin (lower left) and Ker-CT keratinocytes (lower center) produce histologically similar epithelium, while Ker-CT-Ras keratinocytes (lower right) lack the uniformity in the lower strata, although the stratum facing the air retained the ability to cornify. Scale bar: 30 µm.

Ker-CT cells in skin equivalents produced a layered, compartmentalized epithelium (Fig. 2, upper left) similar to normal human skin [12]. With Ker-CT-Ras cells, the epithelium appeared to invade into the dermal compartment (Fig. 2, upper right). At higher magnification, the layering and cell shapes seen in human skin epidermis (Fig. 2, lower left) were almost identical to the Ker-CT epidermis (Fig. 2, lower center). Similarly, in Ker-CT-Ras epidermis (Fig. 2, lower right), cuboidal cells were observed in the lower epidermis, while squamous cells were found near the upper surface epidermis containing a thin cornified layer (Fig. 2, lower right). These upper surface epidermis cellular features of Ker-CT-Ras cells were also present in Ker-CT and normal human skin. There was no apparent invasion by the Ker-CT keratinocytes infected with the control pBABE-hygro vector (data not shown).

**Figure 3:**
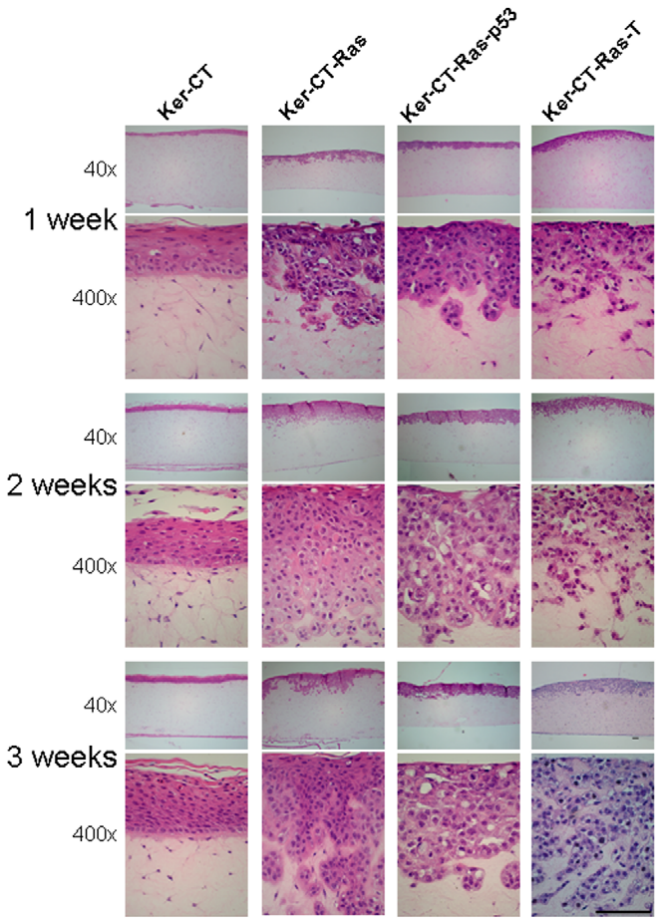
Effect of time on epithelialization. All skin equivalents demonstrated a stratified epithelium on the upper surface within one week. With H-Ras overexpression keratinocyte invasiveness typically occurred early and slowed with time. Similar results occurred with all H-Ras permutations. All but Ker-CT-Ras-T retained some ability to cornify. Upper images, 40× total magnification; lower images, 400× total magnification. Scale bar: 100 µm.

Keratinocytes were seeded as confluent monolayers atop the skin equivalents. At one week of emerged culture, the keratinocytes had proliferated to form a stratified epithelium (Fig. 3, top row). Ker-CT skin equivalents formed a cornified layer by 3 weeks of culture (Fig. 3, bottom left). Dermal invasion of Ker-CT-Ras appeared after one week of emerged culture, the epithelium depth appeared greatest after two weeks, and the cornified layer was most evident after three weeks of emerged culture (Fig. 3, left center). With each H-Ras cell type used, invasion occurred, primarily within the first two weeks, and little subsequent invasion occurred on the third week (Fig. 3). Cornified layering was present by 14 days in Ker-CT-Ras, diminished in Ker-CT-Ras-p53, and was absent in Ker-CT-Ras-T (Fig. 3). These results demonstrate that ectopic expression of H-Ras affects the integrity of the tissue structure, primarily in the lower layers of the epidermis.

### Basement Membrane Staining Is Discontinuous and Diffuse in the Presence of H-Ras Keratinocytes

**Figure 4:**
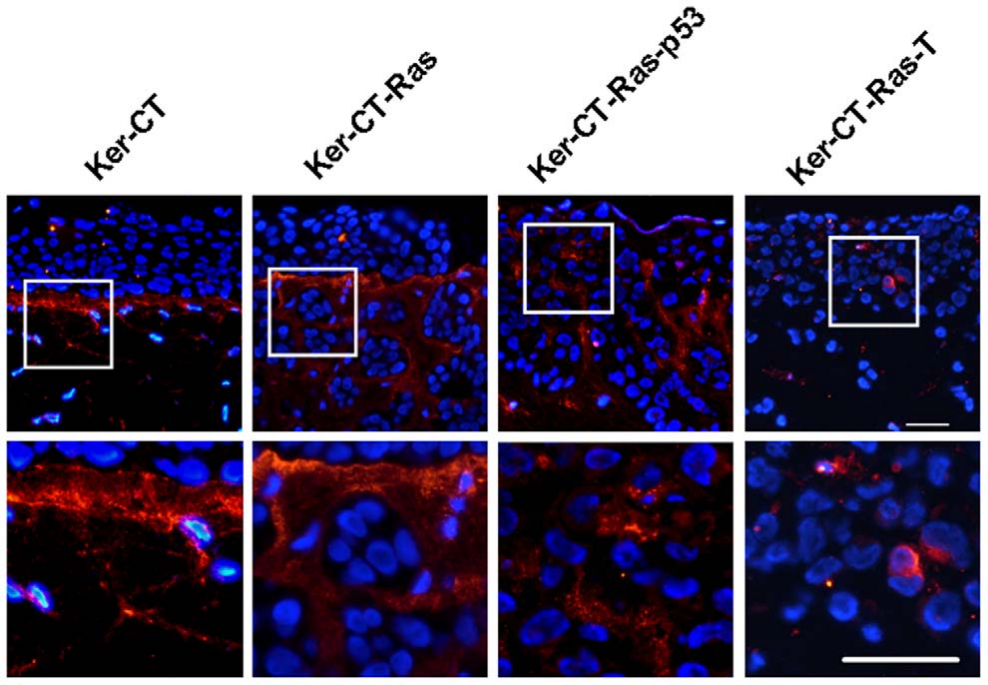
Collagen IV staining in 21-day skin equivalents. Collagen IV staining (red) in Ker-CT skin equivalents increased during the 21 days of emerged culture. Non-linear collagen IV staining appeared in skin equivalents with H-Ras keratinocytes except for Ker-CT-Ras-T, which had little or no collagen IV staining. Lower images are insets of corresponding upper images. Scale bar: 40 µm.

**Figure 5:**
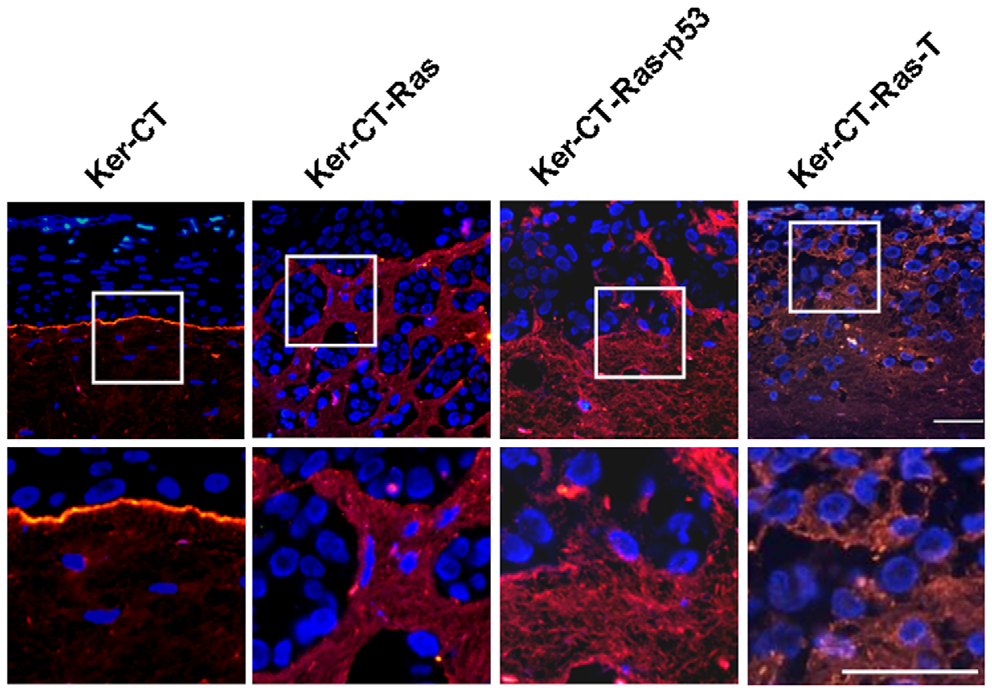
Laminin-5 staining in 21-day skin equivalents. Laminin-5 staining (red) in Ker-CT demonstrated a similar linear staining pattern as Collagen IV. In H-Ras keratinocytes, laminin-5 staining was more prevalent than collagen IV. Lower images are insets of corresponding upper images. Scale bar: 40 µm.

The basement membrane is an important dermal-epidermal landmark that requires contributions from both keratinocytes and fibroblasts [19]–[20]. Since the dermal/epidermal border was indistinct in the Ker-CT-Ras skin equivalents, we next determined whether the deposition of basement membrane proteins would be affected by the altered dermal/epidermal border. We used immunostaining against two basement membrane proteins, collagen IV and laminin-5 to determine the effect of H-Ras on basement membrane formation. Skin equivalents with Ker-CT keratinocytes produced a linear collagen IV staining pattern along the basement membrane zone, increasing with time (Fig. 4, left) similar to our previously-described results [12]. In Ker-CT-Ras skin equivalents a slightly disconnected, diffuse collagen IV staining pattern was observed (Fig. 4, left center). This disconnected basement membrane staining pattern was more pronounced in the Ker-CT-Ras-p53 skin equivalents (Fig. 4, right center). There was little or no staining in Ker-CT-Ras-T skin equivalents (Fig. 4, right). Laminin-5 staining was linear in the Ker-CT cells (similar to the collagen IV pattern), but laminin-5 staining was disorganized in equivalents containing Ker-CT-Ras, Ker-CT-Ras-p53 and Ker-CT-Ras-T cells (Fig. 5). In the Ker-CT-Ras-T skin equivalents the laminin-5 staining was diffuse and adjacent to nearly every epidermal cell. These results suggest that basement membrane proteins are being expressed, but their placement and concentration is affected by the presence of H-Ras keratinocytes.

### Cellular Differentiation Profile Is Maintained by H-Ras Keratinocytes but Begins to Deviate with Subsequent Experimental Modifications

**Figure 6:**
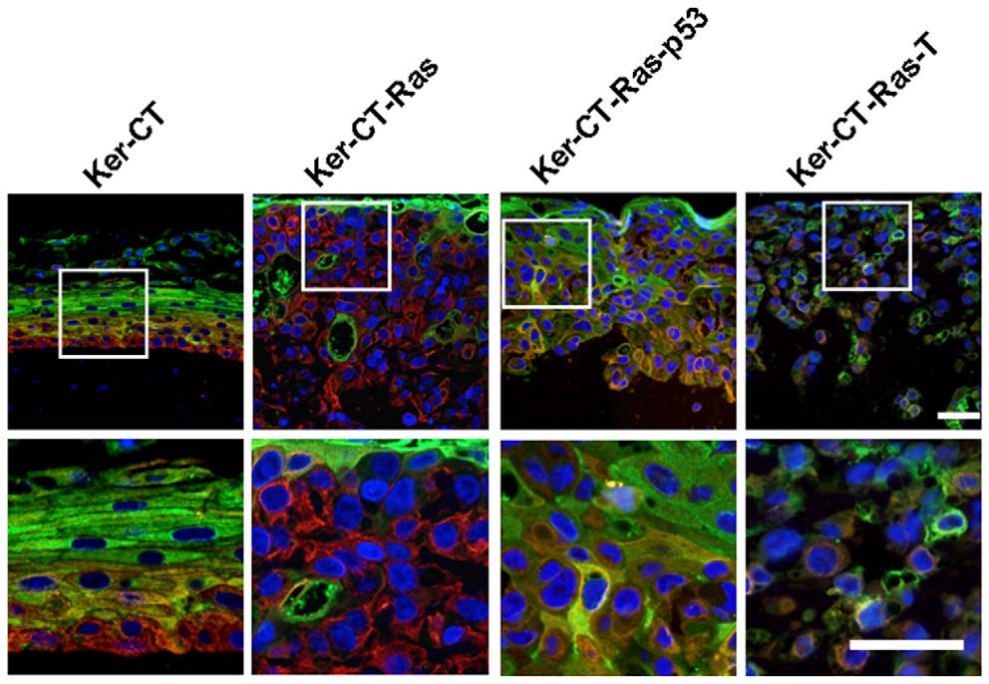
Double-staining of keratin-14 and involucrin in 21-day skin equivalents. In Ker-CT keratinocytes keratin-14 (red) stained the lowermost layers, while involucrin (green) stained the uppermost layers. There was some evidence of co-localization (yellow to orange) in the intermediate layers. Upper staining of involucrin and lower staining of keratin-14 was partially retained in Ker-CT-Ras keratinocytes, but less so in Ker-CT-Ras-p53 keratinocytes. In Ker-CT-Ras-T keratinocytes there was little or no organization of the respective stains. In all tested cells, however, cellular co-localization of both proteins was rare. Lower images are insets of corresponding upper images. Scale bar: 40 µm.

Since the cornified layer was present in H-Ras keratinocytes but the basal layer was disrupted, we next asked whether the layer-specific protein-staining profile was affected by H-Ras expression. We used double-staining of keratin-14 (red fluorescence) and involucrin (green fluorescence) since these proteins represent specific strata of normal epidermis [21]–[22]. In Ker-CT epidermis, keratin-14 staining was present primarily in the basal layer, but not the most superficial layers; involucrin staining was present in the cornified layer and nucleated layers immediately beneath, but not in the basal layer. Some co-localization was seen in the suprabasal layers (Fig. 6, left, yellow/orange color). In Ker-CT-Ras epithelium there was little or no co-localization staining, suggesting that most cells expressed either keratin-14 or involucrin but not both (Fig. 6, left center). In addition, the cells nearest the surface stained intensely for involucrin. Throughout the remainder of the epithelium the majority of the cells stained for keratin-14 with isolated clusters or individual cells staining positive for involucrin. In Ker-CT-Ras –p53 (Fig. 6, right center) and Ker-CT-Ras-T skin equivalents (Fig. 6, right) the surface involucrin staining decreased and the co-localization staining increased. These results suggest that Ker-CT-Ras keratinocytes maintain their differentiation expression profile irrespective of their randomized location, but that subsequent modifications alter the cellular expression profile.

### Orderly Staining of E-Cadherin Is Disrupted in H-Ras Keratinocytes

**Figure 7:**
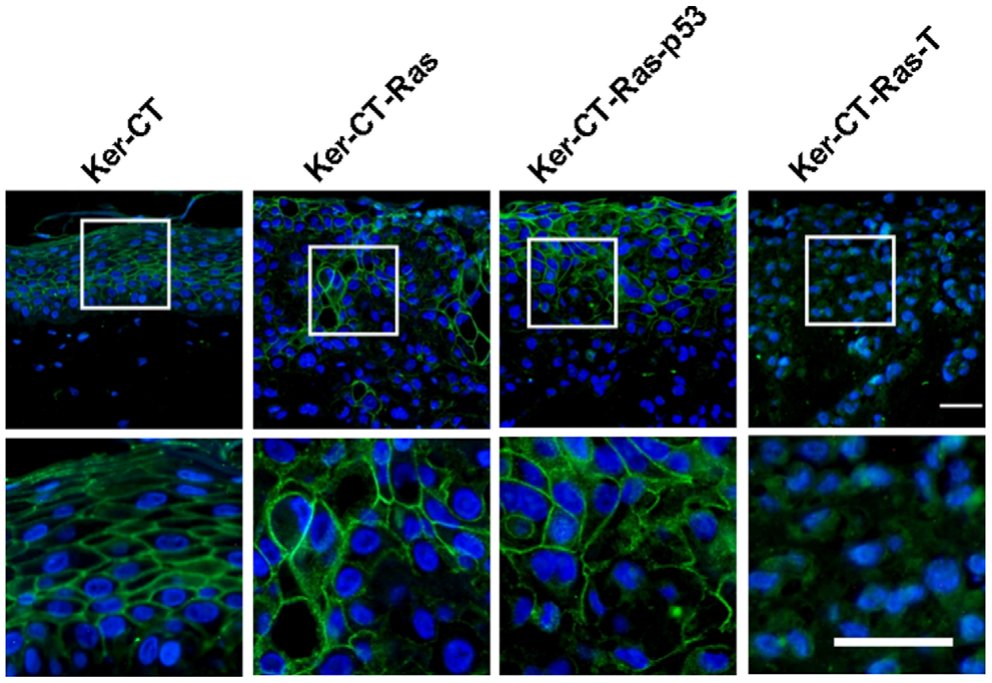
E-cadherin staining in 21-day skin equivalents. E-cadherin staining (green) in Ker-CT keratinocytes demonstrated the cellular connections reminiscent of stratum spinosum in human skin. Little staining was present on the basal surface of the basal layer. E-cadherin staining in H-Ras keratinocytes was less organized than Ker-CT although most of the interconnected cells were in the upper regions of the tissue. Ker-CT-Ras-T had little or no specific E-cadherin staining. Lower images are insets of corresponding upper images. Scale bar: 40 µm.

The integrity of stratified epithelium is maintained in part by their intercellular connections, such as adherens junctions and desmosomes, characterized by E-cadherin presence [23]. Since the histological results suggested that tissue integrity was compromised by H-Ras, especially Ker-CT-Ras-T, we asked whether E-cadherin staining would be similarly affected. In Ker-CT skin equivalents, E-cadherin stained intensely and regularly in the suprabasal layers, but not in the basal layer or the most superficial layer (Fig. 7, left). In Ker-CT-Ras and Ker-CT-Ras-p53 skin equivalents, intense E-cadherin staining was primarily confined to the upper layers, but isolated groups of cells in other areas also stained for E-cadherin (Fig. 7, left center and right center). There was little or no E-cadherin staining in Ker-CT-Ras-T epithelium, which was consistent with the lack of tissue structure demonstrated by Ker-CT-Ras-T in previous figures. These results suggest that E-cadherin absence correlates with loss of tissue integrity due to H-Ras expression.

### Vimentin Expression in Normal Keratinocytes and Ras-Expressing Leratinocytes Is Not Sufficient to Determine EMT but Instead Correlates with Activation

**Figure 8:**
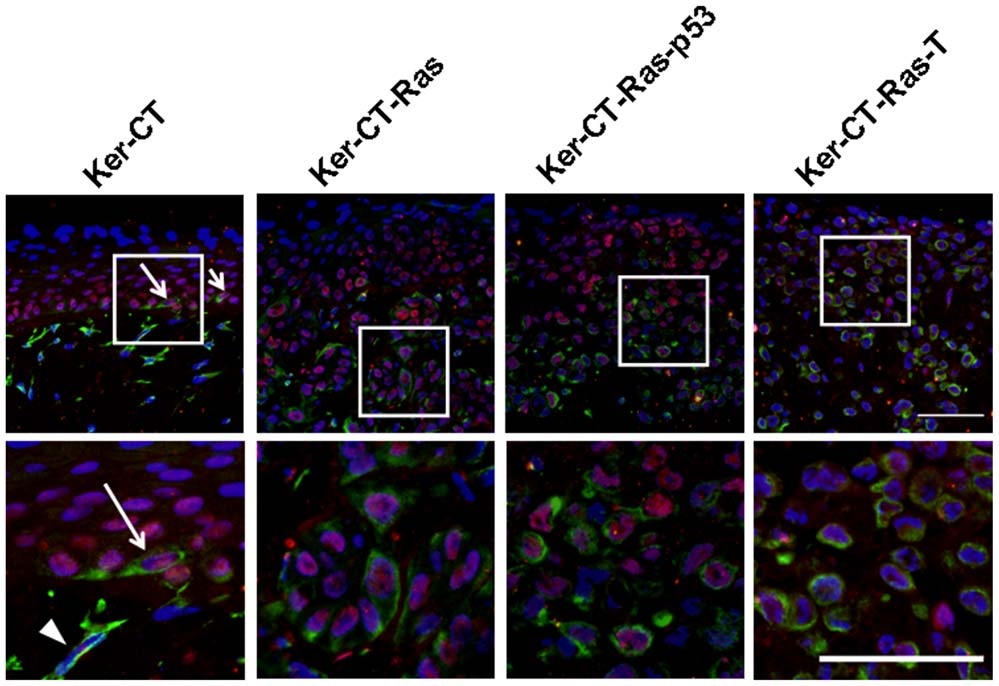
Double-staining of vimentin and p63 in 21-day skin equivalents. p63 (red or pink) and vimentin (green) rarely co-localized in Ker-CT keratinocytes, except in occasional basal cells (arrow). Fibroblasts (arrowhead) were identified by the blue nucleus and green cytoplasm. More co-localization was seen in H-Ras keratinocytes, although primarily in the lower strata. Note the lack of either staining in superficial strata. Lower images are insets of corresponding upper images. Scale bar: 40 µm.

Vimentin, an intermediate filament typically expressed in mesenchymal cells [24], is also present in E6/E7 immortalized keratinocytes [25], as well as metastatic [26], activated [27]–[29], and invasive [30] epithelia. Since the H-Ras cells appeared to be invading the dermal compartment we asked whether they were acquiring vimentin staining as part of the EMT. While Ker-CT skin equivalents only demonstrated dermal staining with vimentin, Ker-CT-Ras skin equivalents demonstrated positive vimentin staining in the epithelial layers (data not shown). To determine whether fibroblasts or keratinocytes were involved, we used immunofluorescence double-labeling of vimentin and p63 to determine if co-localization of both stains occurred. Since p63 is a transcription factor specific to basal or stem cell nuclei of stratified epithelia [31]–[32] we used this protein to detect basal keratinocytes that might have acquired vimentin staining. In Ker-CT skin equivalents, p63 staining was confined to the epithelium and vimentin staining was confined to the dermis (Fig. 8, left). However, an occasional basal-layer keratinocyte stained positive for both proteins (arrows in Fig. 8, left inset), clearly different than the vimentin-staining, bipolar fibroblasts of the dermis (arrowhead in Fig. 8, left inset). In Ker-CT-Ras skin equivalents, more cells demonstrated staining for both p63 and vimentin; in Ker-CT-Ras-p53 and Ker-CT-Ras-T skin equivalents, a substantial number of cells demonstrated co-localization. In all cases the p63-positive, vimentin-positive, and double-staining cells were rarely observed in the upper regions of the epidermis. In addition, the vimentin staining in keratinocytes revealed a cuboidal cell shape that was markedly different than the spindle or bipolar shape of the fibroblasts. These results provide additional evidence that H-Ras expression results in a higher amount of activated or transformed keratinocytes, primarily associated with the lower strata of the skin equivalents.

## Discussion

The goal of this study was to determine whether H-Ras expression in immortalized keratinocytes affected the normal stratification and differentiation of keratinocytes placed in organotypic 3D culture. First, we demonstrated that cdk4 and hTERT immortalized keratinocytes terminally differentiated normally and by all morphological and biochemical criteria were similar to unimmortalized keratinocytes used in skin equivalents. This provided a stable keratinocyte cell line reagent for additional cancer progression experiments. Ras expression in combination with cdk4 and hTERT was sufficient to disorganize the lower strata of the developing epidermis. This was apparent by the presence of involucrin-staining cells surrounded by keratin-14-staining cells.

Interestingly, the combination of cdk4, hTERT, and Ras was insufficient to inhibit terminal differentiation of keratinocytes; the skin equivalents produced a stratified squamous epithelium that cornified in three weeks; the squamous cells lost p63 expression and gained involucrin staining similarly to the control and immortalized keratinocytes. Ras effects on keratinocytes were similar to keratinocyte activation except that the keratinocytes migrated into rather than across the collagen matrix. This, in turn, affected the basement membrane structure, producing a diffuse, incomplete basement membrane.

In keratinocytes with cdk4, hTERT, Ras, and p53 mutant expression, terminal differentiation of keratinocytes was inhibited. There was reduced involucrin staining at the surface and little or no cornification after 3 weeks. There were more cells present that double-stained for involucrin and keratin-14 as well. Finally, the tumorigenic keratinocytes had little tissue organization or appropriate protein expression. There was no cornification, no apparent E-cadherin staining, and little basement membrane staining. Cells migrated into the matrix as individuals rather than groups.

The tumor-cell isolates responded differently to organotypic culture than the Ker-CT-Ras cells, primarily in their attachment to one another. Since multiple genetic modifications in a short timespan might be considered rare, a single genetic modification probably occurred to elicit this response, in the e-cadherin pathway or an alternate that directly affects e-cadherin. But at what point would this have occurred? One possibility is that xenografting enriched for a minor constituent of the Ker-CT-Ras population that was unable to compete in vitro. This hypothesis could be tested by mixing the Ker-CT-Ras-T cells with Ker-CT-Ras cells, culturing them for a specific time period, then placing them into organotypic culture; these cultures might demonstrate the Ker-CT-Ras phenotype, Ker-CT-Ras-T phenotype, or a mixture of the two; a Ker-CT-Ras phenotype under these conditions would suggest that xenografting may help Ker-CT-Ras-T cells outcompete Ker-CT-Ras cells.

An alternate hypothesis is that the xenografting procedure caused the phenotype switch. One way to test this hypothesis would be to culture Ker-CT-Ras cells in the presence of mouse serum, atop mouse subcutaneous tissue, or both, in vitro. This would be followed with removal, blocking, or antagonism of various components in subsequent experiments.

**Figure 9:**
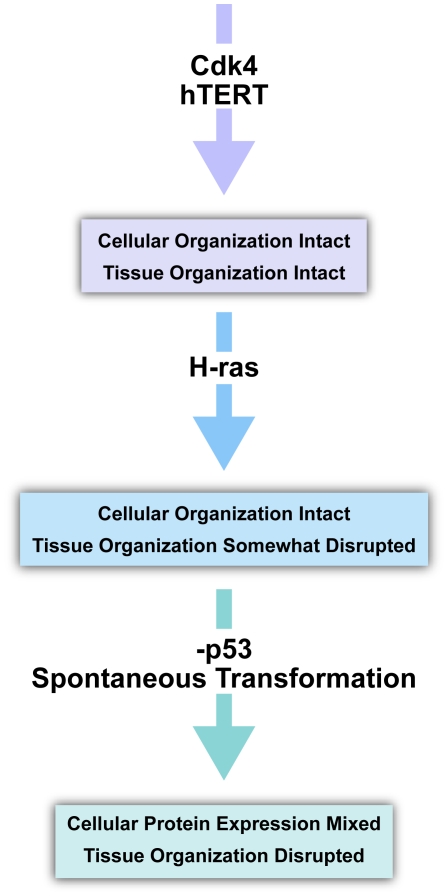
Combined analysis of protein overexpression effects on epithelial integrity. CDK4 and hTERT expression in keratinocytes demonstrate little or no effect to overall tissue integrity or cellular protein staining profile. Upon expression of H-Ras the tissue integrity is affected but the cellular staining profile remains similar to cells without H-Ras expression. After p53 inhibition and transformation, both tissue integrity and cellular identity is lost. Tissue integrity loss may be due to the inappropriate environment itself.

A flow diagram summarizes the course of cellular and tissue transformation events based on these results (Fig. 9). In this diagram, proteins such as cdk4 and hTERT play a passive role in transformation, simply allowing cell division to occur [33]. With H-Ras expression, keratinocyte activation and the disorganization of the tissue environment occur, thereby disrupting the ordered layers of the lower epidermis. Lastly p53 inhibition alters the differentiation pathway that develops the stratum corneum. Subsequent modifications leading to loss of E-cadherin and gain of vimentin expression appear to be necessary for true transformation and malignancy.

It is important to note the limitations of this model. For example, in clinical manifestation of skin cancer a rare or small subset of transformed cells invades into the intact basement membrane of a normal skin structure. In our current series of experiments all cells plated were already transformed and were in direct contact with the fibroblast-populated collagen matrix and exhibited EMT. This model could be modified by mixing only a few transformed keratinocytes with normal keratinocytes in varying ratios to determine if similar results are observed or if there is a suppressive effect of the normal keratinocytes. The inability of Ker-CT cells to invade suggests that Ker-CT-Ras cells are precancerous, with the potential to invade during wound healing or in aging skin where MMPs may be elevated [34]. In both cases, the normal basement membrane would be compromised. It is arguable that our model initially lacking a basement membrane renders it less than optimum to study skin cancer in nonwounded or younger skin. Studies are underway to develop a more appropriate skin cancer model where a basement membrane or basement membrane-like structure is present from the onset of keratinocyte seeding. Such a model might inhibit Ker-CT-Ras invasion, as previously demonstrated using HaCaT-Ras keratinocytes [35].

This study and others [36]–[38] demonstrate that many changes are required to transform a normal keratinocyte into a tumorigenic cell. The environment itself may also play a role in how transformation is manifested [35], [39], [40]. Perhaps the most intriguing result from this study involves the co-localization of p63 and vimentin, suggesting that basal keratinocytes primarily are being activated. These are specifically the cells that include the interfollicular stem cells that appear to have the highest potential for tumorigenesis since their ability to expand is great [41]. Whether any of these cells are interfollicular stem cells or simply transit amplifying cells will require further investigation to identify stem cell markers such as Lrig1 [42]. Continued work with simplified three-dimensional skin cultures is likely to further clarify the fundamental and intricate cellular changes that occur during cancer progression and should be a useful experimental model for chemical and radiation exposures that can affect the skin.

## Materials and Methods

### Cell Culture and Retroviral Infections

All culture methods and protocols used in these experiments were performed and IRB-approved at the University of Texas Southwestern Medical Center. Keratinocytes originally harvested from human foreskin were engineered to ectopically express two proteins (cdk4 and hTERT) to allow continuous culture (Ker-CT cells; see Table 1). Previous studies showed no abnormal effects on growth and differentiation of Ker-CT [12] and these provide for stable diploid non transformed cell reagents for the cancer progression studies. These keratinocytes were infected with a retroviral vector (pBABE-hygro alone or pBABE hygro containing H-Ras (Ker-CT-Ras) in the presence of polybrene [12]. Ker-CT-Ras-T cells were isolated from mouse tumors implanted with Ker-CT-Ras cells. Antibiotic selection in hygromycin was done to remove any contaminating mouse cells. Ker-CT-Ras cells were also infected with a mutant p53 (His^273^) retroviral vector pCMV-Neo [43] and designated Ker-CT-Ras-p53. After antibiotic selection the keratinocytes were used in skin equivalents as described below. Keratinocytes were cultured in a 3∶1 mix of DMEM:F12 as previously described [44]–[45] in the absence of feeder layers, on tissue culture-treated plastic dishes and kept in log-phase growth conditions. Four different keratinocyte cell cultures were used in these experiments (Table 1). Normal dermal foreskin fibroblasts (BJ) were kept in log phase growth and used in skin equivalents as described in Figure 1.

### Organotypic Skin Equivalent Procedure

(Fig. 1) Skin equivalents were prepared by combining rat-tail type I collagen with media salts, bovine sera, and sodium bicarbonate to equilibrate the pH and tonicity [15]; a chilled fibroblast/media mixture (∼2 ml) containing 1.3×10^6^ cells was added to the collagen solution and mixed briefly. 1 ml was then added to each well of a 12-well plate and incubated for 1 hour to polymerize into a lattice. 1 ml of media was added and the lattices were released and allowed to contract for until 90% of the contraction had occurred [16]. After removing media, keratinocytes (2×10^5^ cells/cm^2^) were pipetted into a cloning ring (Bellco Glass) mounted on the upper surface of the contracted lattices, and allowed to attach for 4 hours at 37°C. The ring was removed, and the lattices were submerged 4 days in keratinocyte media with ascorbic acid, then raised to the air/liquid interface (also called emerged culture) using a nontreated culture insert (Transwell; Corning/Costar). Skin equivalents were harvested at indicated times, fixed in 4% paraformaldehyde or 10% neutral buffered formalin overnight at 4°C, washed, then processed for paraffin sectioning. Sections ∼4 µm thick were made with a Leica 2030 microtome (Belair Instrument Company) and mounted onto Superfrost Plus slides (Fisher Scientific). For each cell type reported, at least 2 experiments were performed with duplicate specimens (n = 4).

### Histology, Immunohistochemistry (IHC), and Immunofluorescence (IF)

IHC was performed using a kit as per the manufacturer's instructions (Vectastain; Vector Labs). All primary antibodies were purchased from LabVision (Thermo Fisher) except Laminin-5 (Chemicon). Dewaxing and antigen retrieval prior to immunofluorescence staining on paraffin sections was performed as described [46] with the following modifications: sections were dewaxed at 65°C for 30 minutes, then placed directly into xylene for 15 minutes, followed by another 15-minute xylene treatment. Both IHC and IF were done for all stains on Ker-CT-Ras tissues and demonstrated similar staining patterns; all stains done on remaining tissues were performed using IF only. Secondary fluorescent antibodies used for IF were either goat anti-mouse rhodamine red or goat anti-mouse Alexa 488 (Invitrogen Molecular Probes). Nuclear counterstaining was done by incorporating DAPI (Invitrogen) into the 80% glycerol mounting medium at a concentration of 14.3 µM. For double-label stains, the first primary antibody was incubated at a low concentration overnight at 4°C (p63 or keratin-14), followed by a rabbit primary antibody (vimentin or involucrin) incubated for one hour at room temperature. Goat anti-mouse and goat anti-rabbit secondary antibodies were incubated together for one hour at room temperature.
